# Proteomic analysis reveals heat shock protein 70 has a key role in polycythemia Vera

**DOI:** 10.1186/1476-4598-12-142

**Published:** 2013-11-19

**Authors:** Miguel Gallardo, Santiago Barrio, Marisol Fernandez, Alberto Paradela, Alicia Arenas, Oscar Toldos, Rosa Ayala, Enriqueta Albizua, Ana Jimenez, Santiago Redondo, Rosa Maria Garcia-Martin, Florinda Gilsanz, Juan Pablo Albar, Joaquin Martinez-Lopez

**Affiliations:** 1 Hematology Service, Hospital Universitario 12 de Octubre, Avenida, Córdoba, s/n, 28041, Madrid, Spain; 2 Department of Leukemia, The University of Texas M.D. Anderson Cancer Center, Houston, TX 77030, USA; 3 Proteomics Facility, Centro Nacional de Biotecnología-CSIC, Madrid, Spain; 4 Pathology service, Hospital Universitario 12 de Octubre, Madrid, Spain; 5 Hematology Service, Hospital Virgen de la Salud, Toledo, Spain; 6 Hematology Service, Nuestra Señora de Sonsoles, Avila, Spain

**Keywords:** Polycythemia vera, Essential thrombocythemia, HSP70, 2D-DIGE/MS

## Abstract

JAK-STAT signaling through the JAK2^V617F^ mutation is central to the pathogenesis of myeloproliferative neoplasms (MPN). However, other events could precede the JAK2 mutation. The aim of this study is to analyze the phenotypic divergence between polycytemia vera (PV) and essential thrombocytemia (ET) to find novel therapeutics targets by a proteomic and functional approach to identify alternative routes to JAK2 activation. Through 2D-DIGE and mass spectrometry of granulocyte protein from 20 MPN samples, showed differential expression of HSP70 in PV and ET besides other 60 proteins. Immunohistochemistry of 46 MPN bone marrow samples confirmed HSP70 expression. The median of positive granulocytes was 80% in PV (SD 35%) vs. 23% in ET (SD 34.25%). In an *ex vivo* model *KNK437* was used as an inhibition model assay of HSP70, showed dose-dependent inhibition of cell growth and burst formation unit erythroid (BFU-E) in PV and ET, increased apoptosis in the erythroid lineage, and decreased pJAK2 signaling, as well as a specific siRNA for HSP70. These data suggest a key role for HSP70 in proliferation and survival of the erythroid lineage in PV, and may represent a potential therapeutic target in MPN, especially in PV.

## Introduction

Myeloproliferative neoplasms (MPNs) BCR-ABL negative are clonal, stem cell diseases. Although JAK2 kinase (*JAK2*^*V617F*^*)* is the most frequent mutation it is not the primary molecular event in this group of diseases and several other mutations are described [1-4]. In general these mutations produce an increase in signaling pathways downstream of JAK2. For example, STAT3/5 [5-7] is a central event in the pathogenesis of polycythemia vera (PV). Current treatments only control the symptoms of the disease and do not offer the possibility of a clinical/molecular remission or cure [8,9]. JAK2 inhibitors are emerging as promising new treatments in this disease. However, they do not seem to achieve complete molecular or clinical remission [10,11].

Proteomic screening methods to find new physiopathogenic candidate proteins have not been widely employed in cancer, although a large number of molecular genetic tests have been performed with variable results. One such proteomic method is two-dimensional difference gel electrophoresis (2D-DIGE), which assesses the protein profile in an accessible, economical, and high-resolution manner. However, several studies show that the resolution power of 2D-DIGE decreases when the cellular type or the amount and quality of the protein samples are not selected properly [12].

Molecular chaperones are essential for stabilizing the fragile structures of many receptors, protein kinases, and transcription factors that participate in the pathways of normal cellular growth. Heat shock proteins (HSP) are required to maintain signaling proteins in an active conformation that can be rapidly triggered by growth signals. Thus, HSP may be viewed as facilitators of real-time responses to extracellular signals, particularly in development and cell renewal. Recently, the chaperone HSP90 has been implicated in protection of JAK2 from degradation in the MPN. Thus, the HSP90 inhibitor, PU-H71, has been proposed as an alternative treatment to JAK2 inhibitors [13]. Heat shock protein 70 (HSP70) is related to HSP90 and blocks the apoptotic pathway at different levels [14,15]. HSP70 reduces caspase activation (mainly caspase-3) and suppresses mitochondrial damage and nuclear fragmentation. One of the final targets of caspase-3 is the transcription factor GATA-1 [16,17].

Overexpression of HSP70 can provide a selective survival advantage to tumor cells in part due to its ability to inhibit multiple pathways of cell death, including both intrinsic and extrinsic apoptosis. With regard to the intrinsic apoptosis pathway, HSP70 can bind directly to the pro-apoptotic BCL2 family member BAX and prevent it from translocating to mitochondria, where the latter disrupts mitochondrial membranes following an apoptotic stimulus. Additionally, interaction with HSP70 prevents the recruitment of APAF-1 and procaspase-9 to the apoptosome.

Additionally, HSP70 modulates proliferative pathways via MAPK; it modulates JNK and, RAF-1 and ERK phosphorylation [18-20] HSP70 and HSP90 share the ability to inhibit APAF-1 to block the apoptosis cascade, [21] and it is tempting to speculate a major role of HSP70 and HSP90 in the apoptotic resistance of MPN. These proteins may work separately or together as a HSP90-HOP-HSP70 complex [22,23]. The aim of the present study was to analyze the phenotypic divergence between PV and ET using proteomic screening, with the goal to identify additionally routes to JAK2 inhibitors for targeted therapy. We identified 65 differentially expressed proteins, with HSP70 the most significantly enhanced. HSP70 differential expression was validated by protein expression analysis and an *ex vivo* model of MPN.

## Materials and methods

### Patients

Sixty-seven patients diagnosed with MPN (28 PV, 25 ET JAK2^V617F^, and 14 ET JAK2^WT^) were included in this study, in addition to 26 healthy donors. A diagnosis of MPN was based on the World Health Organization criteria 2001/2008, or the Polycythemia Vera Southern Study Group (according to the standard criteria at the moment of diagnosis). Mutational Screening for JAK2 V617F was performed using real time PCR on DNA from whole peripheral blood.

**Figure 1:**
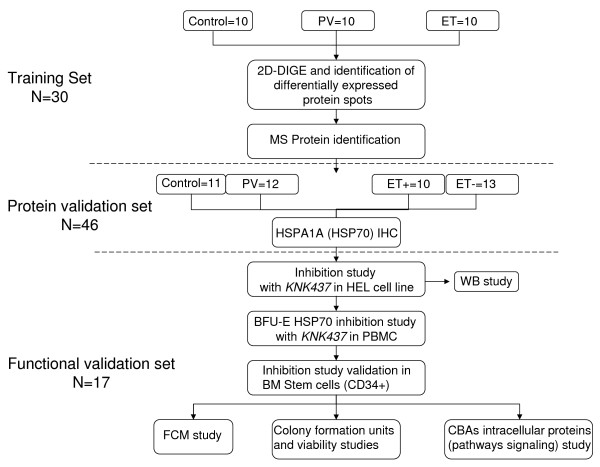
**Flow chart describing the three-steps to proteomic screening for differentially expressed proteins in polycythemia vera (PV) and essential thrombocythemia (ET).** The training set includes two-dimensional difference gel electrophoresis (2D-DIGE) and mass spectrometry (MS); the protein validation set includes immunohistochemistry (IHC); and the functional validation set includes burst formation unit-erythroid (BFU-E) and stem cells studies.

The study was approved by the 12 Octubre Hospital ethics committee and written informed consent was obtained from all patients, according to the Declaration of Helsinki. A flow diagram of the patients is shown in Figure 1.

### Sample collection and preparation

Peripheral venous blood was collected in ethylenediaminetetraacetic acid or heparin lithium and processed immediately. Leukocytes, granulocytes, and mononuclear cells were isolated by Ficoll-Paque density gradient centrifugation (Pharmacia, Freiburg, Germany). Erythrocytes were eliminated using a commercial red blood cell lysis buffer (Roche Applied Sciences, Manheim, Germany), with more than 90% granulocytes. Lymphocyte contamination was assessed in five samples by flow cytometry, and was less than 2% of the total cell count.

Protein cytosolic fractions of granulocytes were extracted using Proteoextract subcellular proteome extraction (Calbiochem, Darmstaz, Germany).

### Determining total protein content

To ensure equal protein loading on both 2D-PAGE and, the protein concentration was determined using a non-interfering assay (RC-DC Protein Assay Kit; BIO-RAD, NY, USA).

### Two-dimensional difference gel electrophoresis

Protein cytosolic fractions from peripheral blood granulocytes from 10 ET, 10 PV, and 10 healthy donors as controls, were initially pooled for two-dimensional difference gel electrophoresis **(**2D-DIGE). We performed three analysis, ET versus PV pools, ET versus control pools, and PV versus control pools by duplicate (6 gels), changing labels with different dye (Cy3 or Cy5), for each pool in each analysis. Patients and clinical data of the 2D-DIGE study are presented in Table 1.

**Table 1:** Clinical and laboratory features of myeloproliferative neoplasm (MPN) patients used for two-dimensional difference gel electrophoresis (2D-DIGE) and mass spectrometry (MS) assays

**Clinical and laboratory variables**	**PV**	**ET**
Patients	10	10
Gender (M/F)	5/5	3/7
Age at diagnosis^a^	68 (60–84)	38 (34–56)
Splenomegaly at diagnosis	0/10	1/10
Hepatomegaly at diagnosis	0/10	1/10
Hemoglobin (g/100 mL) at diagnosis^a^	18.3 (17.4-20.3)	14.95 (12.6-16.9)
Hematocrit at diagnosis^a^	58.25 (51–62.5)	45,52 (37.4-49,7)
WBC (x10^9^/L) at diagnosis^a^	10.65 (3.94-16.15)	12.1 (8.1-14.5)
Platelets (x10^9^/L) at diagnosis^a^	371 (168–394)	615.5 (564–657)
Increased LDH at diagnosis	3/10	1/10
Thrombotic events at diagnosis	1/10	0/10
Hemorrhagic events at diagnosis	0/10	0/10
Thrombotic events after diagnosis	1/10	0/10
Hemorrhagic events after diagnosis	0/10	0/10
Response to treatment	3/6	1/10
Disease duration (in months)	55.5 (14–179)	92.5 (13–100)
Treatment duration (in months)^a^	7 (0–179)	0 (0–86)

PV, polycythemia vera; ET, essential thrombocythemia; HU, hydroxyurea; M, male; F, female; WBC, white blood cells, LDH, lactate dehydrogenase; ^a^Median value (range) is reported.

The PV and ET cytosolic protein pools (20 micrograms each) were minimally labeled with 160 pmol of the N-hydroxysuccinimide esters of Cy3 or Cy5 fluorescent cyanine dyes (GE Healthcare, Piscataway, NJ, USA). An internal standard pool was generated by mixing equal amounts of proteins obtained from all the samples and labeled with 160 pmol of Cy2 dye. PV and ET labeled protein pools and the internal standard protein samples, were combined in pairs, diluted In rehydration buffer (7 M urea, 2 M thiourea, 4% CHAPS, 10 mM DTT, 0.5% carrier ampholites pH 3–11 NL), and applied by cup loading to 18 cm IPG strips pH 3–11 NL (GE Healthcare, Piscataway, NJ, USA), previously rehydrated with 340 μl of rehydration buffer containing 1.2% DeStreak. The first dimension was run at 0.05 mA/IPG strip in an IPGphor IEF System (GE Healthcare, Piscataway, NJ, USA) following a voltage increase until 43000 Vhrs were reached. Strips were then reduced and alkylated in the dark in SDS equilibration buffer (75 mM Tris pH 8,8, 6 M urea, 30% (v/v) glycerol, 2% (w/v) SDS, and traces of bromophenol blue) containing 1% (w/v) DTT or 4% (w/v) iodoacetamide. Finally, the proteins were separated using 12.5% tris-glycine gels in an Ettan Dalt Six device (GE Healthcare, Piscataway, NJ, USA) at 20°C.

### Image acquisition and statistical analysis

Following electrophoresis, the 2D-gels were scanned in a Typhoon 9400 scanner (GE Healthcare, Piscataway, NJ, USA) at 100 μm resolution, and with the appropriate wavelengths and filters for Cy2, Cy3 and Cy5 dyes. Relative protein quantification was performed using DeCyder software v7.0. Background subtraction, quantification, and normalization were automatically applied with low experimental variation. Differences were calculated as average ratios for each spot, and average ratios > or = 1.5 or < or = −1.5. The student’s t-test was used to compare average ratios for each spot between PV and ET samples. P values less than 0.05 were considered significant. Individual coordinates corresponding to the spots of interest were automatically calculated and automatic spot pick-up was carried out using a Spot Picking Robot (GE Healthcare, Piscataway, NJ, USA).

### Protein identification by mass spectrometry

#### In-gel protein digestion and sample preparation

Spots of interest were excised from gels, deposited in 96-well plates and processed automatically in a Proteineer DP (Bruker Daltonics, Bremen, Germany). The digestion protocol used was based on that of Schevchenko *et al.* with minor variations [24]. Modified porcine trypsin (sequencing grade; Promega, Madison, WI) was added at a final concentration of 16 ng/μl in 25% ACN/50 mM ammonium bicarbonate solution and gels were digested at 37°C for 6 h. The reaction was stopped by adding 0.5% TFA for peptide extraction. Tryptic peptides were dried by speed-vacuum centrifugation and resuspended in 4 μl for MALDI TOF/TOF analysis.

### MALDI peptide mass fingerprinting, MS/MS analysis and database searching

For MALDI-TOF/TOF analysis, samples were automatically acquired in an ABi 4800 MALDI TOF/TOF mass spectrometer (AB Sciex, Framingham, MA, USA) in positive ion reflector mode (ion acceleration voltage was 25 kV for MS acquisition and 1 kV for MSMS). PMF and MSMS fragment ion spectra were smoothed, corrected to zero baseline and internally calibrated with the mass signals of trypsin autolysis ions to reach a typical mass measurement accuracy of < 25 ppm. Known trypsin and keratin mass signals, as well as potential sodium and potassium adducts (+21 Da and +39 Da) were removed from the peak list. To submit the combined PMF and MS/MS data to MASCOT software v.2.1 (Matrix Science, London, UK), GPS Explorer v4.9 was used, searching in the non-redundant UniProt/SwissProt protein database (taxonomy: Homo sapiens).

### Immunohistochemistry

Twelve PV, 10 ET JAK2 positive, 13 ET JAK2 negative and 11 controls from formalin fixed and paraffin-embedded bone marrow biopsies were collected. Non haematological diseases patients or patients with secondary thrombocytosis and/or erythrocytosis, both with free-infiltrate bone marrow were used as negative MPN controls. They were used to validate the DIGE/MS results. Patients and clinical data of the IHC study are presented in Table 2.

**Table 2:** Clinical and laboratory features of myeloproliferative neoplasm (MPN) patients used for immunohistochemistry (IHC)

**Clinical and laboratory variables**	**PV**	**ET JAK2 +**	**ET JAK2 -**
Patients	12	10	13
Gender (M/F)	6/12	2/8	7/6
Age at diagnosis^a^	66 (37–72)	55 (33–77)	44 (25–62)
Splenomegaly at diagnosis	2/12	5/10	1/13
Hepatomegaly at diagnosis	2/12	2/10	1/13
Hemoglobin (g/100 mL) at diagnosis^a^	20.05 (17.5-22.5)	15.2 (12.7-16.2)	14.15 (12.1-16.3)
Hematocrit at diagnosis^a^	58 (51.1-66.4)	45.2 (38.3-48.3)	41.15 (35.6-48.9)
WBC (x10^9^/L) at diagnosis^a^	11.1 (5.91-14.7)	8.125 (5.96-11.4)	11.6 (5.24-15.3)
Platelets (x10^9^/L) at diagnosis^a^	436.5 (305–1328)	680 (551–1210)	875 (514–3500)
Increased LDH at diagnosis	4/12	0/10	2/13
Thrombotic events at diagnosis	2/12	1/10	2/13
Hemorrhagic events at diagnosis	0/12	0/10	0/13
Thrombotic events after diagnosis	1/12	1/10	2/13
Hemorrhagic events after diagnosis	1/12	0/10	1/13
Disease duration (in months)	87 (24–138)	148.5 (46–333)	87 (0–256)
Treatment duration (in months)^a^	83 (0–138)	108 (0–330)	87 (0–256)
Response to treatment	8/12	8/10	8/13

PV, polycythemia vera; ET, essential thrombocythemia; HU, hydroxyurea; M, male; F, female; WBC, white blood cells, LDH, lactate dehydrogenase ^a^Median value (range) is reported.

We performed immunohistochemical (IHC) staining in four micron-thick tissue sections from all cohorts for HSP70 (clone MAB1663 anti-h/m/r/rHSP70, 1/8000 dilution; R&D, Minneapolis, MN, USA), SERPINB1 (clone HPA018871 anti-SERPINB1, 1/12000 dilution, SIGMA, Steinheim, Germany), and LTA4H (clone HPA00399 anti-LTA4H, 1/50 dilution, SIGMA, Steinheim, Germany). After incubation, immunodetection was done with the DAKO EnVision visualization method (Dako, Glostrup, Denmark), with diaminobenzidine chromogen as the substrate. Sections were counter stained with hematoxylin. Immunostaining was evaluated by two different pathologists, using granulocyte percent and stain intensity criteria. Only distinct and intense cytoplasmic staining was considered positive.

### Burst formation unit-erythroid culture colony assay

Colony assays were performed using Methocult TM GF_H4535 (StemCell Technologies, Vancouver, BC, Canada, http://www.stemcell.com).

In brief, a 0.5 mL cell suspension, containing 5×10^5^ peripheral blood mononuclear cells from four PV and four ET patients, and three healthy donors as controls, were each mixed in 500 μl of methylcellulose solution consisting of methocult, 20 ng/mL interleukin 3 (IL-3), and 50 ng/mL stem-cell factor (SCF) and 3 U/mL erythropoietin (all fromStemCell Technologies, Vancouver, BC, Canada) in 3.5-cm culture dishes. We cultured the cells with (3 U/mL erythropoietin) and without EPO (endogenous growth).

Additionally, the burst formation unit-erythroid (BFU-E) assay was performed with 2×10^3^ CD34+ bone marrow cells per well from two PV, two ET, and two cord blood samples as controls.

HSP70 was inhibited by 100 μM, 50 μM, and 10 μM *KNK437* (Calbiochem, Darmstadt, Germany). For experimental controls we excluded *KNK437*, and all samples were assayed in duplicate. After 2 weeks, the colonies were counted. Colony morphology was also observed using an inverted light microscope.

Cells from the BFU-E were extracted, washed and resuspended in 10 mL PBS. Ten microlitres aliquots of cells were used to test viability using trypan blue (1:1). Cells were analyzed by flow cytometry after the addition of 10 μl (5 μl of APC antibodies) of markers; CD71-FITC, CD45-PerCP, CD44-PE, annexin V-APC, CD41a-FITC, and CD34-APC (BD Biosciences Europe, Oxford, UK), incubated for 30 minutes at 4°C, and washed with PBS or binding buffer 1X (BD Biosciences Europe, Oxford, UK) (in the case of annexin) before analysis. Samples were analyzed using a flow cytometer FACSCalibur (Beckman Coulter, Fullerton, CA). Cell suspensions with IgG isotype control antibodies (BD Biosciences Europe, Oxford, UK) were used as negative controls.

DNA from BFU-E cultured cells was extracted using the Maxwell 16 SEV automated extraction system (Promega, Manheim, Germany).

Protein from cells was extracted with the CBA extraction kit (BD Biosciences Europe, Oxford, UK) according to the manufacturer’s instructions but with the addition of phosphatase and protease inhibitors. CBA technology is a set of microspheres (beads) with different sizes and fluorescent intensities and each bead binds a specific protein (similar to the ELISA technique). Each CBA assay includes seven principal steps: preparation of beads, preparation of Phycoerythrin reagent, setting standard curve, preparation of samples, cytometer calibration, acquisition of samples, and file analysis. We analyzed four phosphorylated, and their respective native, proteins: AKT, p-ATK, P38, p-P38, MEK, p-MEK, STAT1, and p-STAT1. These proteins represent the most important pathways downstream of the JAK2 signaling pathway. Protein concentrations were analyzed using concentration ratios of phosphoproteins normalized with non-phosphoproteins and total protein.

### KNK437 dose–response curve on HEL and Ba/F3 JAK2 V617F EPOR cell lines culture

To confirm the above CBA results, we analyzed JAK-STAT and MAPK activation after *KNK437* treatment, a specific pharmacological HSP70 inhibitor, in HEL and Ba/F3 JAK2 V617F EPOR cell lines that were kindly transferred by Dr A. Quintas-Cardama for MD Anderson, and cultured as previously described [25]. We used these cell lines as MPN model due to its JAK2 mutational status. HEL cells were obtained from the DSMZ collection (cells were obtained on 01/08/2008; JAK2 V617F mutation status was tested by QRT-PCR on 01/06/2012, finding JAK2 V617F mutation in homocigosis) and cultured in RPMI-1640 medium containing 10% fetal calf serum, with L-glutamine and NaHCO_3_ in a humidified 5% CO_2_ atmosphere. For the inhibition assay, subconfluent cells in 9.5 cm^2^ wells (P6) were treated with *KNK437* (50 μM) for 24 hours. Results were analyzed with the trypan blue viability test. Cells were washed twice in PBS and protein was extracted with the Cytobuster protein extraction reagent (Novagen, EMD Bioscences Inc., Madision, WI). The protein concentration was determined using a non-interfering assay (RC-DC Protein Assay Kit; BIO-RAD, NY, USA) and Western Blot was performed using rabbit anti-actin primary antibody (protein control), anti-p-MEK (ser217/221), anti-ERK, anti-p-ERK (thr202/tyr204), anti-p-P38 (thr180/tyr182), anti-JAK2, anti-p-JAK2 (tyr1007/1008) , anti-STAT5, anti-p-STAT5 (ser456) (Cell signaling, Beverly, MA, USA), and mouse anti-HSP90 and anti-HSP70 (R&D, Minneapolis, MN, USA). The membranes were then incubated with the respective secondary antibodies for 1 h and antigens were detected by using the ECL Advance Western Blotting Detection Kit (GE Healthcare, Piscataway, NJ, USA).

### HSP70 interference on HEL cell line culture

In order to confirm the specificity of KNK437 over HSP70, we analyze the effect of the interference on HSP70 molecule through a specific siRNA. HEL cell line was transfected using the Amansa Electronucleofector 2b and Cell Line Nucleofector kit V (Lonza, NJ, USA). Anti HSP70 siRNA Trilencer 27 was acquired from Origene (Rockvile, MD, USA). Cells were incubated 8 h. Pmax gfp (Lonza USA, NJ) was used as a fluorescent control, showing a transfection efficacy greater than 80%.

#### Statistical and bioinformatic analysis

The 2D-DIGE results were analyzed with a Batch Processor of DeCyderv7.0 with the following parameters: 1) For PV vs. ET analysis an increase or diminution of 1.5 times and t-test P < 0.05 was considered significant. In addition, the spot should be found in all extracted images. 2) For ET vs. healthy donors, and PV vs. healthy donors, parameters were an increase or diminution of 3 times, and t-test P < 0.01. We apply a higher cutoff due to larger differences found compared to PV vs ET analysis, in order to select a small number of spots for further studies.

Mass spectrometry results were analyzed with Mascot software.

Western blot data images were analyzed using ImageJ (National Institute of Health, USA) with gel tool. Numerical data were processed with the Mann–Whitney test (SPSS, SPSS Inc., Chicago, Illinois, USA).

Flow cytometry was run in Cell Quest software, and data were analyzed with the Summit 4.3 program. Extracted numerical data were analyzed statistically with the Mann–Whitney test (SPSS, SPSS Inc., Chicago, Illinois, USA).

IHC and culture data were also analyzed using the Mann–Whitney test.

*KNK437* Ic50 of BFU-E inhibition assays were calculated using the GraphPathPrism 5.04 (GraphPath Software Inc.).

CBAs files were processed with FCAP Array™ (BD Biosciences Europe, Oxford, UK), and data analyzed with the Mann–Whitney test.

Statistical significance was considered when the P-value was < 0.05.

## Results

### Identification of differentially expressed proteins using Two-dimensional difference gel electrophoresis and mass spectrometry

**Figure 2:**
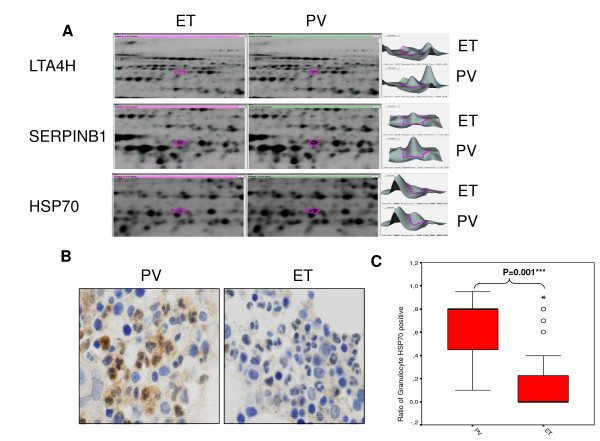
**Two-dimensional difference gel electrophoresis (2D-DIGE) analysis and IHC validation. (A)** Representative spots from three proteins from 2D-DIGE gels and intensity quantification with DeCyder software v7.0. three spots were identified as LTA4H, SERPINB1 and HSP70 (HSPA1A). These spots were over-expressed in polycythemia vera (PV) vs. essential thrombocythemia (ET) samples. **(B)** Representative images according the median of HSP70 staining of granulocytes from bone marrow of PV, and ET. **(C)** Box-plot of percentage of HSP70 positive granulocytes quantified from IHC.

DIGE and MS were used to identify differences in the whole cytosolic proteome between PV and ET groups. Figure 2A, show three representative spots from the proteomic analyses of samples from ET and PV patients. We found 112 spots representing proteins with differential expression between both diseases. Identification of the spots yielded 65 proteins. Three proteins were especially interesting in the context of our model and selected for further studies by doing a literature search on their biological function. These three differentially expressed proteins includes LTA4H, SERPINB1 and HSP70 (Figure 2A). Of note, HSP70 is a chaperone related to GATA-1 and erythroid differentiation. Most of the other spots corresponded to a large group of proteins implicated in metabolic and biochemical processes, for example, glycogen phosphorylase, pyruvate kinase, and lactotransferrin. Healthy donors also showed differences when compared with PV samples. There were 174 spots and 19 proteins identified (HSP70 included). Samples from controls and ET showed differences in 97 spots, and six proteins were identified. Most of the proteins identified were implicated in metabolic and biochemical pathways, similarly to those observed when ET and PV were compared. A full list of the differentially expressed proteins is summarized in Additional file 1: Table S1, Additional file 2: Table S2 and Additional file 3: Table S3.

### Validation of proteomic analysis by immunohistochemistry in bone marrow

Bone marrow IHC analysis of HSP70, SERPINB1, and LTA4H was performed to confirm and identify the expression pattern found by 2D-DIGE/MS. Twelve bone marrow biopsies from PV patients, 10 ET JAK2 V617F positive, 13 ET JAK2 V617F negative, and 11 controls were studied. HSP70 was markedly over-expressed in PV bone marrow (median of 80% positive granulocytes, range 2-95%, SD 0.35%) vs. ET (median of 23% positive granulocytes, range 1-95%, SD 34.25%) (Figure 2B-C). A full list of the HSP70 expression sample per sample is summarized in Additional file 4: Table S4.

SERPINB1 was expressed in the nucleus and cytoplasm of the granulocytes. We found a differential expression pattern between the bone marrow of controls (median of 20% positive granulocytes, range 2-99%, SD 39.05%) and PV (median of 95% positive granulocytes, range 65-99%, SD 16.72%), ET JAK2 V617F (median of 67% positive granulocytes, range 5-99%, SD 36.04%), and ET JAK2 wild-type (median of 98% positive granulocytes, range 3-98%, SD 38.31%).

LTA4H also was expressed in granulocytes and in a small percent of the megakaryocytic lineages. It was expressed in 90-100% of the bone marrow studied and in 90-100% of the granulocytes, and no differences were observed between the different groups (data not shown).

### HSP70 inhibition ex-vivo study; implication in polycythemia vera erythroid differentiation

Inhibition of HSP70 with *KNK437* showed similar results in primary BFU-E cultures, with and without EPO. Bone marrow CD34+ cell cultures showed equivalent results to peripheral blood mononuclear cell cultures.

**Figure 3:**
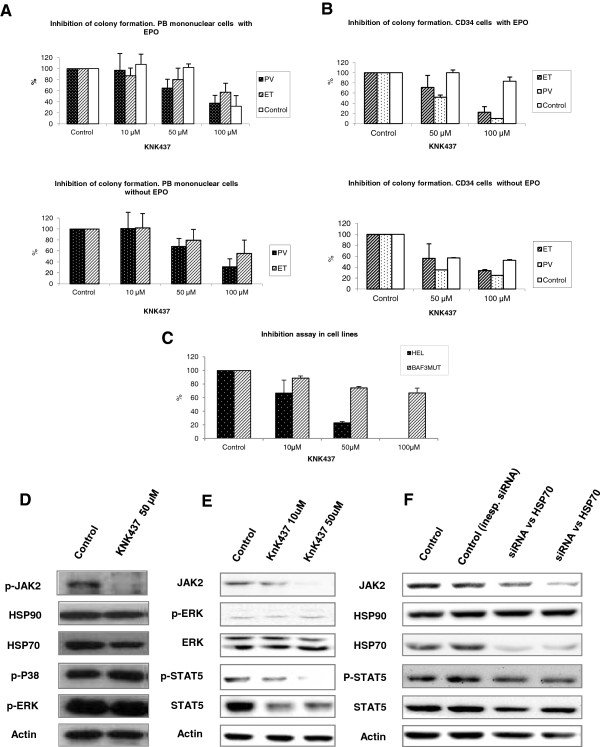
***KNK437 *inhibition assay of cell cultures. (A)** Viability test results of *KNK437* (10, 50 and 100 μM) inhibition assay of mononuclear peripheral blood burst formation unit-erythroid (BFU-E) cultures, with and without EPO. **(B)** Viability test results of a *KNK437* (50 and 100 μM) inhibition assay of CD34+ bone marrow BFU-E cultures, with and without EPO. **(C)** Viability test results of *KNK437* treatment (50 μM) on HEL and Ba/F3 JAK2 V617F (BAF3MUT) cell lines. X axis: concentration (μM); Y axis: % of viability cells (viability test) or BFU-E (BFU-E cultures) **(D)** Molecular effects of KNK437 in HEL cell line Western blot of HSP90, HSP70, p-JAK2 (decreased with *KNK437* treatment), p-ERK and p-P38. Actin was used as the housekeeping control. **(E)** Molecular effects of KNK437 in Ba/F3 cell line Western blot of JAK2, p-ERK, ERK, p-STAT5, STAT5 AND Actin, **(F)** Western blot of HSP90, HSP70 , JAK2, STAT5 and p-STAT5. Expression levels of HSP70, JAK2 and p-STAT5 decreased after HSP70 siRNA interference on HEL cell line. Actin was used as the housekeeping control.

BFU-E cultures of CD34+ cells with *KNK437* showed a decrease of colony formation and erythroid precursor viability. This *KNK437*-mediated decrease of viability reached an IC_50_ of 20.05 μM in PV samples. Erythroid precursor cell viability in cord blood samples and ET patient cells was higher (IC_50_ = 38.87 μM) than in the PV patients (Figure 3A-B). *KNK437* also decreased cell viability in the HEL and Ba/F3 JAK2 V617F cell lines (Figure 3C). However, statistical significance among the groups was not found.

**Figure 4:**
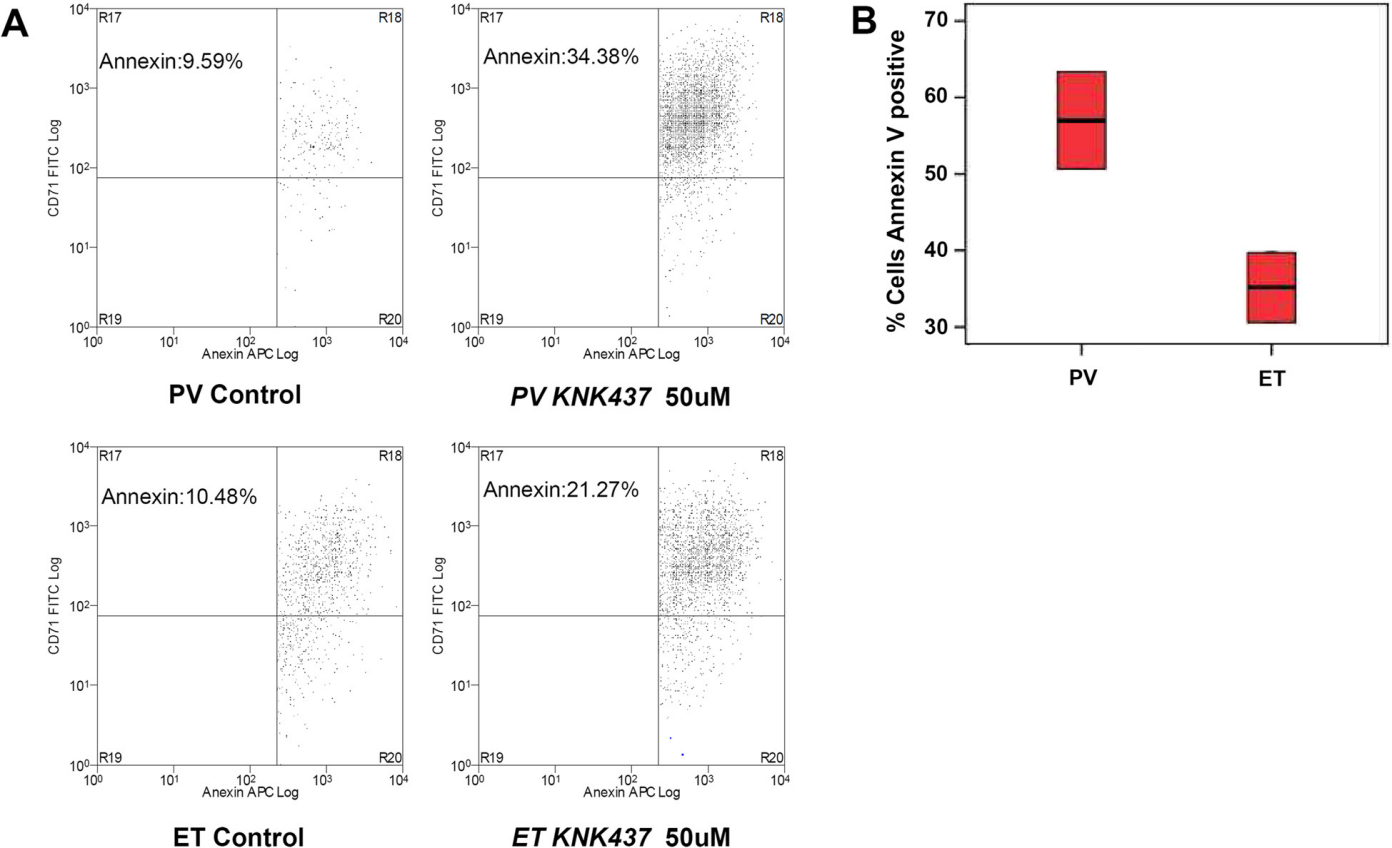
**Flow cytometry of *KNK437 *inhibition assay of CD34+ cell cultures. (A)** FCM results of BFU-E cultured cells from CD34+ bone marrow of polycythemia vera (PV) and essential thrombocythemia (ET) samples, with and without *KNK437* treatment (50 μM), and with the CD71 marker (y axis) and annexin V (x axis). **(B)** Box-plot of percentage of Annexin V positive CD34+ culture cells quantified by flow cytometry.

Flow cytometry results of BFU-E colonies showed differences in apoptosis among the erythroid population (that express CD71) in untreated PV cultures (10% annexin positive cells) vs. treated *(KNK437*, 50 μM) cultures (34% annexin positive cells). However, the same differences were not seen when treated ET samples (*KNK437*, 50 μM) (21% annexin positive cells) were compared with untreated cells (10% annexin positive cells). Figure 4A-B shows the flow cytometry results for the CD34+ BFU-E cultures.

CBA analysis showed an important decrease of phospho-STAT1 in PV samples patients (ratio of pSTAT1 in PV without treatment: 29.2; ratio of pSTAT1 in PV samples with *KNK437*, 50 μM: 21.55), however, we found no significant differences in phospho-STAT1 with and without *KNK437* treatment in ET patients. We define ratios as concentration ratios of phosphoproteins normalized with non-phosphoproteins as total protein numeric value. Additionally, phospho-MEK showed under-expression after *KNK437* treatment, and this was more pronounced in samples from PV patients (ratio in PV without treatment: 63.17; ratio in PV with *KNK437*, 50 μM: 23.58) vs. ET patients (ratio in ET without treatment: 66.19; ratio in ET with *KNK437*, 50 μM: 59.82). Moreover, the other MAPK phospho-protein, phospho-p38, was differentially expressed with and without *KNK437* treatment in samples from PV patients (ratio in PV without treatment: 7.30; ratio in PV with *KNK437*, 50 μM: 4.18), but was unchanged in ET patients. Phospho-AKT showed no decrease with treatment. A full list of the proteins and phospho-proteins expression sample per sample is summarized in Additional file 5: Table S5.

### HSP70 inhibition in an ex-vivo cell line

To confirm the molecular mechanism of the HSP70 inhibitor in the JAK2/STAT and MAPK pathways, we performed Western blot on HEL and Ba/F3 JAK2 V617F cell lines proteomes, with and without *KNK437* (10-50-100 μM) treatment (Figure 3D). This showed a reduction of the phospho-JAK2 and phospho-STAT5 protein with treatment (Figure 3E and F), but no reduction of phospho-ERK and phospho-p38. ImageJ quantification confirmed these results and showed a 50% reduction in the expression of phospho-JAK2 in the HEL cell line following treatment with *KNK437*. Additionally, HSP70, HSP90 and detection by Western Blot showed a slight decrease of HSP70 expression after *KNK437* treatment, but no significant difference in HSP90 expression in the HEL cell line, with and without *KNK437* treatment (Figure 3D). KNK437 decreased the activation of JAK2 as well as its expression. This decrease in JAK2 expression resulted in the inhibition of leading proliferative pathways related to JAK2 (Figure 3E) GATA1 also showed no differential expression with the HSP70 inhibitor treatment (Additional file 6: Figure S1). Similarly to the primary BFU-E, incubation with the HSP70 inhibitor *KNK437* (50 μM) in HEL and Ba/F3 JAK2 V617F caused a reduction of 20-50% in the cell viability (Figure 3C).

In order to validate the KNK437 inhibition on HSP70, and check the specificity of this treatment, additional HSP70 interference was performed with specific a siRNA (Figure 3F). The results showed a proper interference, decreasing the protein levels of HSP70, but not HSP90. Besides, HSP70 interference assay produces the decrease of the expression of JAK2, and the inhibition of JAK-STAT signaling due to the decrease of phospho-STAT5.

## Discussion

Many authors believe in the possibility of other events and/or genetic alterations ‘upstream’ of the JAK2 mutation in MPN [26-32]. This opens new frontiers in the pathogenesis of the disease and the phenotypic divergence among the different MPNs must be studied to find new defective molecules that may potentially be used for novel targeted therapies. Proteomic screening to find new molecular targets has been an under-used strategy in MNP. This may be due to several factors, namely the difficultly in selecting the correct target cell populations and their protein fractions, or the lack of a high-quality protein extraction technique. Moreover, these approaches can lead to a huge number of differentially expressed proteins that can introduce confusion in the absence of a proper analysis. These putative differences also need to be confirmed with further, specific, single-protein analyses such as IHC.

In overcoming those problems, 2D-DIGE approach could represent an unexplored and efficient method to find new molecular targets in hematology. We found molecular divergences between PV and ET granulocyte proteins. With 2D-DIGE we found more than sixty differentially expressed proteins when we compared samples from PV and ET patients. We selected three proteins for further studies due to their biological importance: LTA4H, HSP70, and SERPINB1 (Figure 2A-B-C). The LTA4H differences were not confirmed with IHC. SERPINB1, however, was differentially expressed in the controls and all MPN groups. Although the cohorts were small, we could suggest validation of Gel 2D-DIGE technique results, above all HSP70 PV over-expression. However with this data we could just validate previous results with other methodology, neither the use of few number of samples not encourage to use these data to other aim. Based on these results, further studies are needed to elucidate its importance as a MPN biomarker.

We focused on HSP70 expression. Surprisingly, this protein was over-expressed in samples from PV patients compared with ET and healthy donors, and this difference between PV and ET was confirmed with IHC (Figure 2B). This led us to investigate the effect of HSP70 inhibition in an *ex vivo* model of MPN. We demonstrated that *KNK437*, a HSP70 inhibitor, increased erythroid apoptosis in cell cultures from PV patients (Figure 4A-B). This effect could be mediated by JAK2 inhibition, given that a decreased phosphorylation was shown after *KNK437* treatment (Figure 3D). This was corroborated by the decrease of phosphoSTAT1 through cytometric bead array results and over Ba/F3 JAK2V617F cell line (Figure 3E). Additionally, we performed siRNA HSP70 interference assay, observing similar results to KNK437 treatment: an inhibition of JAK-STAT signaling. Thus, the results support the specificity of KNK437, demonstrating that the effect of KNK437 is due to the specific inhibition of HSP70. But more importantly, these observations confirm the role of HSP70 in the pathogenesis of PV, and that it could play a role as a new molecular target for the treatment of this disease.

These data reflect the key implication of HSP70 in PV disease, playing a key role in proliferation, differentiation, and survival of the erythroid lineage. Inactivation of the JAK/STAT pathway by the HSP70 inhibitor may be the explanation. In accordance with the putative importance of HSP in the pathogenesis of JAK-STAT -related hematological disorders, a recent study described the potential therapeutic use of PU-H71, a HSP90 inhibitor, in experimental models of MPN, ET and PV [13]. This study described a crosstalk between JAK2 and a HSP90-like molecule, since HSP90 inhibition was able to decrease JAK2.

Unfortunately, the clinical efficacy of HSP90 inhibitors has been generally disappointing. One possible reason for this is that treatment of cancer cell lines with HSP90 inhibitors generally leads to significant activation of HSF1 and up-regulation of HSP70; indeed, up-regulation of HSP70 is a key biomarker for the inhibition of HSP90. Interestingly, however, it was discovered that HSP70 inhibition ‘alone’ effectively disrupts the HSP90 chaperone system [33].

In the this study, we showed that inhibition of HSP70 decreases JAK2 activation (Figure 3D, E and F). However, we found no significant effect of HSP70 inhibition on HSP90. In particular, HSP70 inhibition by *KNK437 or siRNA* led to a decrease in JAK2 and STAT1 or STAT5 phosphorylation, whereas HSP90 remained unaffected (Figure 3D and E). HSP70 and HSP90 may exert parallel effects in JAK2 activation. Recent experimental data show that they may bind to the HOP protein and thus form a HSP70-HOP-HSP90

In summary, we have demonstrated that HSP70 could be implicated in the pathogenesis of PV by means of a comprehensive translational model from the systematic proteomic analysis of the cytosolic fractions of the granulocytes of PV patients, and we confirmed these results with IHC. Eventual proof of concept of the importance of HSP in this disease was achieved by inhibiting the proliferation/apoptotic ratio and the blockade of JAK/STAT activation in cultured PV patient cells, after incubating these cells with the HSP inhibitor, KNK437 or siRNA. Given the moderate effect of direct, target-designed JAK2 blockers in MPN treatment, [34,35] HSP70 inhibitors, may present a promising future therapeutic strategy for PV patients.

## Competing interest

The authors declare that they have no competing interests.

## Authors’ contributions

MG designed the research, contributed to the conception, collected samples and clinical data, performed the majority of experiments, analyzed and interpreted data, and performed and wrote the manuscript. SB contributed to the conception, collected samples and clinical data, performed experiments and analyzed and interpreted data. MF performed 2D-DIGE gels technique, analyzed and interpreted data and performed and wrote the manuscript. AP performed mass spectrometry, analyzed and interpreted data and performed and wrote the manuscript. OT collected samples and clinical data, and analyzed and interpreted immunohistochemistry data. RA collected samples and clinical data. EA contributed to the conception, collected samples and clinical data. AJ collected samples and clinical data and analyzed and interpreted data. SR collected samples and clinical data, revised the manuscript. RMGM performed immunohistochemistry technique. FG revised clinical data and supervised research and experiments. JPA designed 2D-DIGE and mass spectrometry research and supervised experiments. JML designed the research, contributed to the conception, supervised research and experiments, performed and wrote the manuscript and critically revised the manuscript. All authors reviewed and accepted the manuscript. All authors read and approved the final manuscript.

## Supplementary Material

Additional file 1: Table S1 MALDI-TOF/TOF identification of proteins with significant changes in expression levels in polycythemia vera (PV) and essential thrombocythemia (ET) patients. Click here for file

Additional file 2: Table S2 MALDI-TOF/TOF Identification of proteins with significant changes in levels in ET patients. Click here for file

Additional file 3: Table S3 MALDI-TOF/TOF Identification of proteins with significant changes in levels in PV patients. Click here for file

Additional file 4: Table S4 IHC patients list and HSPA1A % of positive granulocytes. Click here for file

Additional file 5: Table S5 CBA results sample per sample from BFU-E cultures with treatment (KNK437 50 mcM) and without treatment (control). Click here for file

Additional file 6: Figure S1 Western blot of GATA1. GATA1 WB from HEL cell line with and without KNK437 treatment (50 mcM). Actin was used as the housekeeping control. Click here for file

## Statement of originality

The authors confirm that this manuscript contains original material.

## Prior presentations of this study

Gallardo M, Fernandez M, Paradela A, Toldos O, Garcia-Martin R, Barrio S, Ayala R, Albizua E, Rapado I, Jimenez A, Candelas M, Gilsanz F, Martinez-Lopez J. Proteomic analysis identifies HSP70 as a novel target therapy to polycythemia vera. *Blood* (ASH Annual Meeting Abstracts) 2011. Presented at 53th ASH Annual Meeting, San Diego, December 2011.
