# Supplementary material for: Proteomic analysis reveals heat shock protein 70 has a key role in polycythemia Vera
Source: Mol Cancer. 2013 Nov 19;12:142. doi: 10.1186/1476-4598-12-142 (PMC4225507; doi:10.1186/1476-4598-12-142)
Supplement: Additional file 1: Table S1 — MALDI-TOF/TOF identification of proteins with significant changes in expression levels in polycythemia vera (PV) and essential thrombocythemia (ET) patients. [file 1476-4598-12-142-S1.doc]

**Additional file 1:Table S1**

**MALDI-TOF/TOF identification of proteins with significant changes in expression levels in polycythemia vera (PV) and essential thrombocythemia (ET) patients**

| **Sample name^a^** | **Accession code^b^** | **Protein description** | **DeCyder P value (t-test)** | **Average ratio^c^** | **MASCOT score** | **Theoretical MW (Da)** | **Theoretical pI** | **% coverage^d^** |  |
| --- | --- | --- | --- | --- | --- | --- | --- | --- | --- |
|  |
| 526 | P06737 | Glycogen phosphorylase, liver form | 7.40E-05 | 1.54 | 427 | 97486 | 6.71 | 35 | geles ≥3 |
| 534 | P06737 | Glycogen phosphorylase, liver form | 1.00E-06 | -1.49 | 432 | 97486 | 6.71 | 32 | geles ≥3 |
| 539 | P06396 | Gelsolin | 4.10E-06 | 1.85 | 186 | 86043 | 5.9 | 23 | geles ≥3 |
| 542 | P06396 | Gelsolin | 0.00031 | 1.41 | 565 | 86043 | 5.9 | 39 | geles ≥3 |
| 555 | P02788 | Lactotransferrin | 1.50E-07 | -2.35 | 828 | 80014 | 8.5 | 61 | geles ≥3 |
| 561 | P06396 | Gelsolin | 9.60E-06 | -1.67 | 667 | 86043 | 5.9 | 40 | geles ≥3 |
| 564 | P02788 | Lactotransferrin | 2.00E-06 | -4.31 | 970 | 80014 | 8.5 | 68 | geles ≥3 |
| 626 | P02788 | Lactotransferrin | 0.0019 | 1.66 | 333 | 80014 | 8.5 | 45 | geles ≥3 |
| 657 | P02788 | Lactotransferrin | 1.40E-06 | -1.56 | 956 | 80014 | 8.5 | 64 | geles ≥3 |
| 661 | P02788 | Lactotransferrin | 2.50E-08 | -2.06 | 670 | 80014 | 8.5 | 52 | geles ≥3 |
| 671 | P26038 | Moesin | 0.00041 | 1.63 | 300 | 67892 | 6.08 | 49 | geles ≥3 |
| 678 | P26038 | Moesin | 3.80E-05 | -1.91 | 203 | 67892 | 6.08 | 38 | geles ≥3 |
| 752 | P29401 | Transketolase | 3.90E-08 | -3.25 | 311 | 68519 | 7.58 | 31 | geles ≥3 |
| 757 | P02788 | Lactotransferrin | 4.70E-06 | -1.85 | 275 | 80014 | 8.5 | 37 | geles ≥3 |
| 800 | P09960 | Leukotriene A-4 hydrolase | 2.30E-05 | 2.44 | 450 | 69868 | 5.8 | 49 | geles ≥3 |
| 803 | P09960 | **Leukotriene A-4 hydrolase** | 4.40E-05 | 1.92 | 727 | 69868 | 5.8 | 60 | geles ≥3 |
| 886 | P14618 | Pyruvate kinase isozymes M1/M2 | 1.50E-08 | -3.08 | 190 | 58470 | 7.96 | 53 | geles ≥3 |
| 892 | P14618 | Pyruvate kinase isozymes M1/M3 | 2.50E-08 | -2.85 | 113 | 58470 | 7.96 | 22 | geles ≥3 |
| 903 | P04040 | Catalase | 1.90E-06 | -1.69 | 742 | 59947 | 6.9 | 56 | geles ≥3 |
| 907 | P04040 | Catalase | 1.30E-05 | 1.62 | 506 | 59947 | 6.9 | 48 | geles ≥3 |
| 917 | P06396 | Gelsolin | 7.50E-06 | 1.53 | 67 | 86043 | 5.9 | 17 | geles ≥3 |
| 939a | P31146 | Coronin-1A | 0.00016 | -7.81 | 140 | 51678 | 6.25 | 33 | geles ≥3 |
| 939b | P13645 | Keratin, type I cytoskeletal 10 | 0.00016 | -7.81 | 150 | 59020 | 5.13 | 31 | geles ≥3 |
| 960 | P06744 | Glucose-6-phosphate isomerase | 5.90E-07 | -2.03 | 212 | 63335 | 8.43 | 43 | geles ≥3 |
| 962 | P06744 | Glucose-6-phosphate isomerase | 9.90E-06 | 1.69 | 248 | 63335 | 8.43 | 39 | geles ≥3 |
| 1038 | P02788 | Lactotransferrin | 6.90E-07 | -1.63 | 497 | 80014 | 8.5 | 35 | geles ≥3 |
| 1104 | P61158 | Actin-related protein 3 | 3.30E-07 | 1.62 | 555 | 47797 | 5.61 | 62 | geles ≥3 |
| 1131 | P52209 | 6-phosphogluconate dehydrogenase, decarboxylating | 2.50E-07 | -1.96 | 198 | 53619 | 6.8 | 22 | geles ≥3 |
| 1152 | P06733 | Alpha-enolase | 5.00E-06 | -2.57 | 441 | 47481 | 7.01 | 59 | geles ≥3 |
| 1160a | P06733 | Alpha-enolase | 0.00022 | 1.56 | 287 | 47481 | 7.01 | 43 | geles ≥3 |
| 1160b | P31146 | Coronin-1A | 0.00022 | 1.56 | 110 | 51678 | 6.25 | 36 | geles ≥3 |
| 1228 | P35527 | Keratin, type I cytoskeletal 9 | 0.00037 | 1.5 | 81 | 62255 | 5.14 | 26 | geles ≥3 |
| 1288 | P00558 | Phosphoglycerate kinase 1 | 0.00019 | 1.78 | 98 | 44985 | 8.3 | 21 | geles ≥3 |
| 1289 | P00558 | Phosphoglycerate kinase 1 | 3.40E-06 | 1.6 | 93 | 44985 | 8.3 | 30 | geles ≥3 |
| 1339 | P08107 | Heat shock 70 kDa protein 1A/1B | 0.0018 | -1.61 | 195 | 70294 | 5.48 | 31 | geles ≥3 |
| 1353a | P09972 | Fructose-bisphosphate aldolase C | 1.80E-06 | 1.56 | 82 | 39830 | 6.41 | 8 | geles ≥3 |
| 1353b | P04075 | Fructose-bisphosphate aldolase A | 1.80E-06 | 1.56 | 57 | 39851 | 8.3 | 26 | geles ≥3 |
| 1364 | P30740 | **Leukocyte elastase inhibitor** | 0.0087 | 1.75 | 423 | 42829 | 5.9 | 35 | geles ≥3 |
| 1478 | P53004 | Biliverdin reductase A | 1.90E-06 | 1.53 | 206 | 33692 | 6.06 | 22 | geles ≥3 |
| 1494 | P04406 | Glyceraldehyde-3-phosphate dehydrogenase | 8.50E-08 | -2.08 | 354 | 36201 | 8.57 | 28 | geles ≥3 |
| 1504 | P04406 | Glyceraldehyde-3-phosphate dehydrogenase | 0.00022 | 1.68 | 364 | 36201 | 8.57 | 45 | geles ≥3 |
| 1515 | P04406 | Glyceraldehyde-3-phosphate dehydrogenase | 6.40E-05 | 1.71 | 151 | 36201 | 8.57 | 33 | geles ≥3 |
| 1635 | P07355 | Annexin A2 | 3.80E-05 | 1.63 | 974 | 38808 | 7.57 | 64 | geles ≥3 |
| 2847 | P35527 | Keratin, type I cytoskeletal 9 | 2.00E-05 | 1.65 | 63 | 62255 | 5.14 | 37 | geles ≥3 |
| 452a | P63261 | Actin, cytoplasmic 2 | 0.016 | -1.71 | 91 | 42108 | 5.31 | 36 | geles =2 |
| 452b | P35527 | Keratin, type I cytoskeletal 9 | 0.016 | -1.71 | 72 | 62255 | 5.14 | 14 | geles =2 |
| 509 | P02788 | Lactotransferrin | 3.30E-05 | 1.82 | 141 | 80014 | 8.5 | 19 | geles =2 |
| 526 | P02788 | Lactotransferrin | 0.015 | 1.5 | 508 | 80014 | 8.5 | 42 | geles =2 |
| 557 | P26038 | Moesin | 0.015 | 1.85 | 230 | 67892 | 6.08 | 34 | geles =2 |
| 558a | P35908 | Keratin, type II cytoskeletal 2 epidermal | 0.00031 | -2.07 | 343 | 65678 | 8.07 | 28 | geles =2 |
| 558b | P04264 | Keratin, type II cytoskeletal 1 | 0.00031 | -2.07 | 226 | 66170 | 8.15 | 25 | geles =2 |
| 558c | P13645 | Keratin, type I cytoskeletal 10 | 0.00031 | -2.07 | 209 | 59020 | 5.13 | 25 | geles =2 |
| 558d | P26038 | Moesin | 0.00031 | -2.07 | 95 | 67892 | 6.08 | 36 | geles =2 |
| 591 | P16050 | Arachidonate 15-lipoxygenase | 0.011 | -1.55 | 219 | 75498 | 6.14 | 28 | geles =2 |
| 603 | P02768 | Serum albumin | 0.0053 | -2.02 | 74 | 71317 | 5.92 | 21 | geles =2 |
| 609 | P29401 | Transketolase | 0.018 | 1.52 | 671 | 68519 | 7.58 | 44 | geles =2 |
| 615 | P07355 | Annexin A2 | 0.0024 | -2.07 | 959 | 38808 | 7.57 | 64 | geles =2 |
| 640 | O75083 | WD repeat-containing protein 1 | 0.0028 | -1.73 | 550 | 66836 | 6.17 | 47 | geles =2 |
| 642 | O75083 | WD repeat-containing protein 1 | 0.00045 | 1.63 | 128 | 66836 | 6.17 | 22 | geles =2 |
| 825 | P06744 | Glucose-6-phosphate isomerase | 0.031 | -1.52 | 572 | 63147 | 8.42 | 32 | geles =2 |
| 972 | P52209 | 6-phosphogluconate dehydrogenase, decarboxylating | 0.0024 | 1.67 | 324 | 53619 | 6.8 | 24 | geles =2 |
| 1134 | P00558 | Phosphoglycerate kinase 1 | 0.0058 | -1.52 | 204 | 44985 | 8.3 | 35 | geles =2 |
| 1158 | P08107 | **Heat shock 70 kDa protein 1A/1B** | 0.0045 | 1.63 | 620 | 70294 | 5.48 | 51 | geles =2 |
| 1165 | P08107 | Heat shock 70 kDa protein 1A/1B | 0.0031 | -1.75 | 471 | 70294 | 5.48 | 40 | geles =2 |
| 1325 | P04083 | Annexin A1 | 0.025 | -1.5 | 88 | 38918 | 6.57 | 45 | geles =2 |

**Additional file 1:Table S1**

**:** ^a^Spot numbering according to location in 2D gels. ^b^Protein accession code (SwissProt/UniProt). ^c^The average ratio value indicates the standardized volume ratio between polycythemia vera (PV) and essential thrombocythemia (ET). If values are < or = -1.5 indicates a decrease in expression in PV and > or = 1.5 indicates an increase in expression in PV. ^d^Percentage of coverage was calculated using the sequence of the full-length protein.
