# Supplementary material for: Proteomic analysis reveals heat shock protein 70 has a key role in polycythemia Vera
Source: Mol Cancer. 2013 Nov 19;12:142. doi: 10.1186/1476-4598-12-142 (PMC4225507; doi:10.1186/1476-4598-12-142)
Supplement: Additional file 2: Table S2 — MALDI-TOF/TOF Identification of proteins with significant changes in levels in ET patients. [file 1476-4598-12-142-S2.doc]

**Additional file 2:Table S2**

**MALDI-TOF/TOF Identification of proteins with significant changes in levels in ET patients.**

| **Sample name^a^** | **Accession code^b^** | **Protein description** | **DeCyder P value (t-test)** | **Average ratio^c^** | **MASCOT score** | **Theoretical MW (Da)** | **Theoretical pI** | **% coverage^d^** |  |
| --- | --- | --- | --- | --- | --- | --- | --- | --- | --- |
|  |
| 545 | P06396 | Gelsolin | 1,20E-07 | 12,77 | 284 | 86043 | 5,9 | 31 | geles ≥3 |
| 639 | P35241 | Radixin | 4,20E-08 | 5,18 | 73 | 68635 | 6,03 | 20 | geles ≥3 |
| 674 | P26038 | Moesin | 2,90E-08 | 7,7 | 153 | 67892 | 6,08 | 26 | geles ≥3 |
| 908 | P14618 | Pyruvate kinase isozymes M1/M2 | 1,80E-09 | 6,48 | 162 | 58470 | 7,96 | 42 | geles ≥3 |
| 958 | P31146 | Coronin-1A | 1,00E-04 | 3,66 | 57 | 51678 | 6,25 | 13 | geles ≥3 |
| 1510 | P04406 | Glyceraldehyde-3-phosphate dehydrogenase | 2,10E-10 | 12,89 | 103 | 36201 | 8,57 | 44 | geles ≥3 |

**Additional file 2:Table S2**

**:** ^a^Spot numbering according to location in 2D gels. ^b^Protein accession code (SwissProt/UniProt). ^c^The average ratio value indicates the standardized volume ratio between control and essential thrombocythemia (ET). If values are < or = -3.0 indicates a decrease in expression in PV and > or = 3.0 indicates an increase in expression in PV. ^d^Percentage of coverage was calculated using the sequence of the full-length protein.
