# Supplementary material for: Proteomic analysis reveals heat shock protein 70 has a key role in polycythemia Vera
Source: Mol Cancer. 2013 Nov 19;12:142. doi: 10.1186/1476-4598-12-142 (PMC4225507; doi:10.1186/1476-4598-12-142)
Supplement: Additional file 3: Table S3 — MALDI-TOF/TOF Identification of proteins with significant changes in levels in PV patients. [file 1476-4598-12-142-S3.doc]

**Additional file 3: Table S3**

**MALDI-TOF/TOF Identification of proteins with significant changes in levels in PV patients.**

| **Sample name^a^** | **Accession code^b^** | **Protein description** | **DeCyder P value (t-test)** | **Average ratio^c^** | **MASCOT score** | **Theoretical MW (Da)** | **Theoretical pI** | **% coverage^d^** |  |
| --- | --- | --- | --- | --- | --- | --- | --- | --- | --- |
|  |
| 545 | P06396 | Gelsolin | 2,00E-07 | 12,16 | 199 | 86043 | 5,9 | 34 | geles ≥3 |
| 629 | P02788 | Lactotransferrin | 8,70E-08 | 3,83 | 92 | 80014 | 8,5 | 26 | geles ≥3 |
| 677 | P26038 | Moesin | 3,90E-07 | 6,44 | 303 | 67892 | 6,08 | 45 | geles ≥3 |
| 680 | P26038 | Moesin | 3,10E-07 | 4,24 | 198 | 67892 | 6,08 | 31 | geles ≥3 |
| 745 | P29401 | Transketolase | 2,90E-09 | 9,66 | 521 | 68519 | 7,58 | 42 | geles ≥3 |
| 755 | P29401 | Transketolase | 1,90E-08 | 5,79 | 718 | 68519 | 7,58 | 48 | geles ≥3 |
| 892a | P14618 | Pyruvate kinase isozymes M1/M2 | 1,10E-07 | 11,61 | 100 | 58470 | 7,96 | 59 | geles ≥3 |
| 892b | P04040 | Catalase | 1,10E-07 | 11,61 | 80 | 59947 | 6,9 | 30 | geles ≥3 |
| 898 | P14618 | Pyruvate kinase isozymes M1/M2 | 4,00E-07 | 5,6 | 110 | 58470 | 7,96 | 53 | geles ≥3 |
| 905 | P04040 | Catalase | 2,90E-06 | 3,01 | 111 | 59947 | 6,9 | 16 | geles ≥3 |
| 906 | P14618 | Pyruvate kinase isozymes M1/M2 | 1,30E-08 | 4,43 | 260 | 58470 | 7,96 | 46 | geles ≥3 |
| 936 | P31146 | Coronin-1A | 6.3-009 | 4,29 | 205 | 51678 | 6,25 | 45 | geles ≥3 |
| 1005 | P11413 | Glucose-6-phosphate 1-dehydrogenase | 3,30E-08 | 3,01 | 208 | 59675 | 6,39 | 36 | geles ≥3 |
| 1028 | P11413 | Glucose-6-phosphate 1-dehydrogenase | 9,70E-07 | 5,88 | 335 | 59675 | 6,39 | 52 | geles ≥3 |
| 1103 | P61158 | Actin-related protein 3 | 2,20E-06 | 3,3 | 98 | 47797 | 5,61 | 30 | geles ≥3 |
| 1162 | P06733 | Alpha-enolase | 3,70E-09 | 4,58 | 58 | 47481 | 7,01 | 31 | geles ≥3 |
| 1163 | P13929 | Beta-enolase | 1,20E-07 | 14,22 | 56 | 47244 | 7,59 | 32 | geles ≥3 |
| 1327 | P09960 | **Leukotriene A-4 hydrolase** | 1,20E-05 | -1,97 | 55 | 69868 | 5,8 | 40 | geles ≥3 |

**Additional file 3: Table S3**

**:** ^a^Spot numbering according to location in 2D gels. ^b^Protein accession code (SwissProt/UniProt). ^c^The average ratio value indicates the standardized volume ratio between control and polycythaemia vera (PV). If values are < or = -3.0 indicates a decrease in expression in PV and > or = 3.0 indicates an increase in expression in PV. ^d^Percentage of coverage was calculated using the sequence of the full-length protein.
