# Supplementary material for: Proteomic analysis reveals heat shock protein 70 has a key role in polycythemia Vera
Source: Mol Cancer. 2013 Nov 19;12:142. doi: 10.1186/1476-4598-12-142 (PMC4225507; doi:10.1186/1476-4598-12-142)
Supplement: Additional file 4: Table S4 — IHC patients list and HSPA1A % of positive granulocytes. [file 1476-4598-12-142-S4.doc]

**Additional file 4:Table S4**

**IHC patients list and HSPA1A % of positive granulocytes**

|  | % positive granulocytes |
| --- | --- |
| PV patient 1 | 70% |
| PV patient 2 | 95% |
| PV patient 3 | Negative |
| PV patient 4 | 2% |
| PV patient 5 | Negative |
| PV patient 6 | 80% |
| PV patient 7 | Negative |
| PV patient 8 | 80% |
| PV patient 9 | 80% |
| PV patient 10 | 80% |
| PV patient 11 | 10% |
| PV patient 12 | 80% |
| **PV median** | **80%** |
| ET patient 1 | 95% |
| ET patient 2 | Negative |
| ET patient 3 | 60% |
| ET patient 4 | 95% |
| ET patient 5 | 3% |
| ET patient 6 | 5% |
| ET patient 7 | 40% |
| ET patient 8 | Negative |
| ET patient 9 | 2% |
| ET patient 10 | 1% |
| ET patient 11 | Negative |
| ET patient 12 | Negative |
| ET patient 13 | 80% |
| ET patient 14 | No valuable |
| ET patient 15 | Negative |
| ET patient 16 | Negative |
| ET patient 17 | 2% |
| ET patient 18 | 1% |
| ET patient 19 | 70% |
| ET patient 20 | 90% |
| ET patient 21 | 2% |
| ET patient 22 | 1% |
| ET patient 23 | 85% |
| **ET median** | **23%** |
| Control patient 1 | 60% |
| Control patient 2 | 90% |
| Control patient 3 | Negative |
| Control patient 4 | Negative |
| Control patient 5 | 70% |
| Control patient 6 | 85% |
| Control patient 7 | 70% |
| Control patient 8 | Negative |
| Control patient 9 | 1% |
| Control patient 10 | 85% |
| Control patient 11 | 95% |
| **Control median** | **78%** |

**Additional file 4:Table S4**

Complete list of patients, PV, ET and Control groups, and percent of HSP70 positive granulocytes over IHC bone marrow biopsies sample per sample and group per group. (Negative: <1%) (No valuable: No stain).
