# Supplementary material for: Proteomic analysis reveals heat shock protein 70 has a key role in polycythemia Vera
Source: Mol Cancer. 2013 Nov 19;12:142. doi: 10.1186/1476-4598-12-142 (PMC4225507; doi:10.1186/1476-4598-12-142)
Supplement: Additional file 5: Table S5 — CBA results sample per sample from BFU-E cultures with treatment (KNK437 50 mcM) and without treatment (control). [file 1476-4598-12-142-S5.doc]

**Additional file 5: Table S5**

**CBA results sample per sample from BFU-E cultures with treatment (KNK437 50 mcM) and without treatment (control).**

| Samples | [ ] total protein | Total P38 | p-P38 | p-MEK | Ratio p-38 | Ratio p-MEK | TotalSTAT1 | p-STAT1 | Ratio p-STAT1 | Total AKT | p-AKT | Ratio p-AKT |
| --- | --- | --- | --- | --- | --- | --- | --- | --- | --- | --- | --- | --- |
| PV1 control | 1,145922747 | 648,38 | 17,11 | 6,3 | 43,42451 | 117,935459 | 797,63 | 10,14 | 90,14027224 | 93,75 | 25,18 | 4,2664916 |
| PV1 KNK437 50 mcM | 0,453505007 | 172,09 | 18,63 | 3,05 | 4,18914 | 25,5880907 | 394,96 | 8,31 | 21,55431259 | 63,36 | 23,8 | 1,2073142 |
| PV2 control | 0,837625179 | 608,76 | 48,58 | 4,67 | 10,49635 | 109,189016 | 614,27 | 13,72 | 37,50204217 | 80,31 | 24,95 | 2,6961795 |
| PV2KNK437 50 mcM | 0,433476395 | 190,1 | 13,59 | 4,67 | 6,063566 | 17,6453667 | 181,69 | 9,23 | 8,532863075 | 42,32 | 23,58 | 0,777978 |
| ET1 control | 1,190271817 | 672,17 | 68,47 | 7,43 | 11,6849 | 107,680351 | 955,02 | 12,7 | 89,50656619 | 128,8 | 28,43 | 5,3924379 |
| ET1 KNK437 50 mcM | 0,444921316 | 170,54 | 9,05 | 3,91 | 8,384186 | 19,405852 | 191,82 | 7,98 | 10,69483795 | 40,79 | 22,71 | 0,7991343 |
| ET2 control | 1,808297568 | 742,18 | 315,2 | 14,96 | 4,25747 | 89,711383 | 722,39 | 18,11 | 72,13120266 | 81,05 | 71,34 | 2,0544227 |
| ET2 KNK437 50 mcM | 0,508583691 | 522,3 | 26,38 | 2,65 | 10,06949 | 100,238967 | 468,76 | 2,65 | 89,96365698 | 83,29 | 22,71 | 1,8652548 |

**Additional file 5: Table S5**

We define ratios as concentration ratios of phosphoproteins normalized with non-phosphoproteins as total protein numeric value.
