# Supplementary material for: Proteomic analysis reveals heat shock protein 70 has a key role in polycythemia Vera
Source: Mol Cancer. 2013 Nov 19;12:142. doi: 10.1186/1476-4598-12-142 (PMC4225507; doi:10.1186/1476-4598-12-142)
Supplement: Additional file 6: Figure S1 — Western blot of GATA1. GATA1 WB from HEL cell line with and without KNK437 treatment (50 mcM). Actin was used as the housekeeping control. [file 1476-4598-12-142-S6.ppt]

## Slide 1
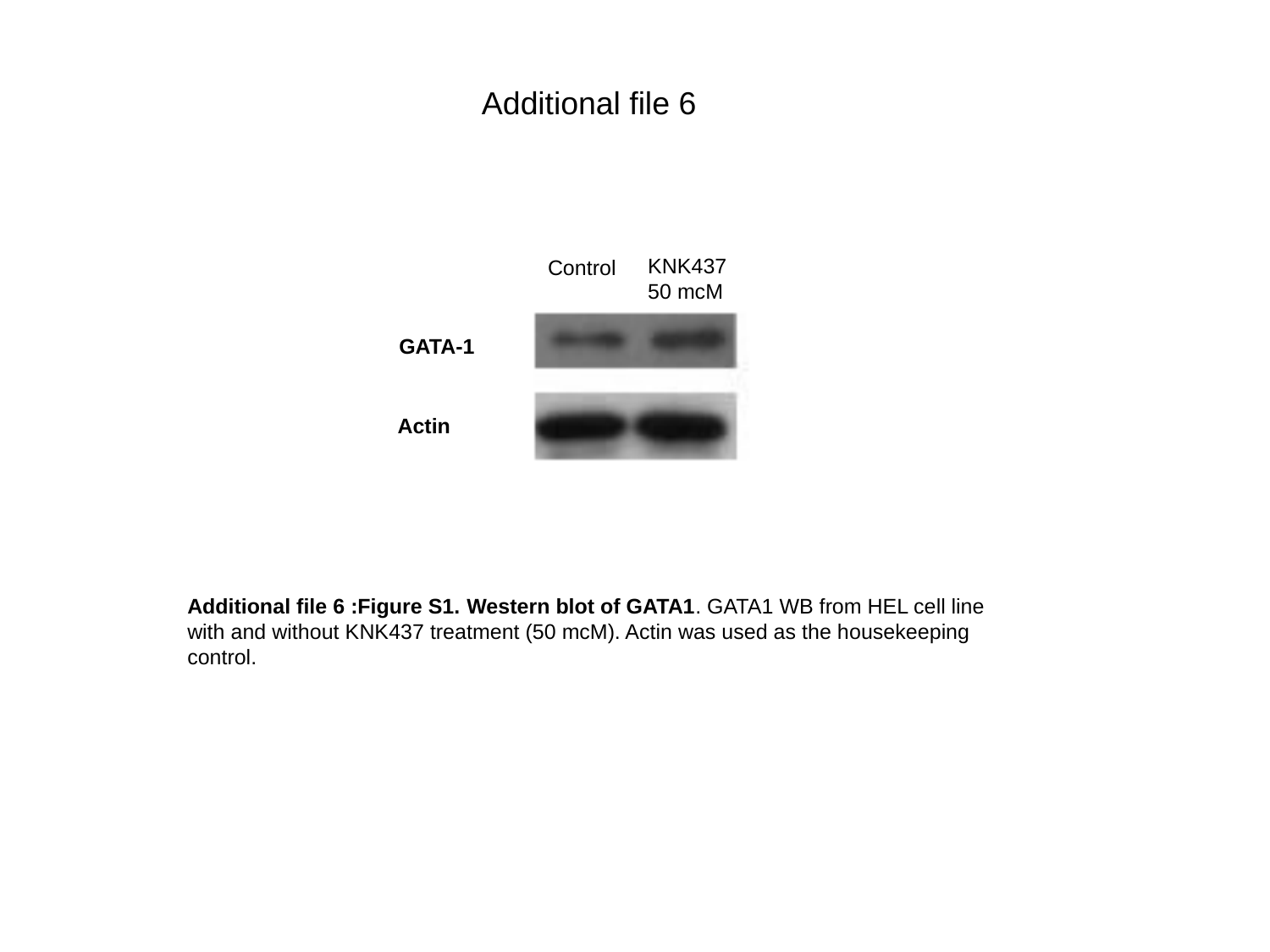

Additional file 6
KNK437
50 mcM
Control
GATA-1
Actin
Additional file 6 :Figure S1. Western blot of GATA1. GATA1 WB from HEL cell line
with and without KNK437 treatment (50 mcM). Actin was used as the housekeeping control.
